# Vascular endothelial growth factor-C expression in human prostatic carcinoma and its relationship to lymph node metastasis

**DOI:** 10.1038/sj.bjc.6690356

**Published:** 1999-04

**Authors:** T Tsurusaki, S Kanda, H Sakai, H Kanetake, Y Saito, K Alitalo, T Koji

**Affiliations:** Departments of Urology, andHistology and Cell Biology, Nagasaki University School of Medicine, 1-7-1 Sakamoto, Nagasaki 852-8102, Japan; Molecular/Cancer Biology Laboratory, Haartman Institute, University of Helsinki, PL21, 00014 Helsinki, Finland

**Keywords:** prostatic carcinoma, vascular endothelial growth factor-C, VEGF receptor-3, lymph node metastasis

## Abstract

Lymph node dissemination is a major prognostic factor in human cancer. However, the molecular mechanisms underlying lymph node metastasis are poorly understood. Recently, vascular endothelial growth factor-C (VEGF-C) was identified as a ligand for VEGF receptor-3 (VEGFR-3/Flt-4) and the expression of VEGFR-3 was found to be highly restricted to the lymphatic endothelial cells. In this report, we investigated the expression of VEGF-C and VEGFR-3 in human prostatic carcinoma tissue by using in situ hybridization and immunohistochemical staining respectively. Expression of VEGF-C mRNA in prostatic carcinoma was significantly higher in lymph node-positive group than in lymph node-negative group. In addition, the number of VEGFR-3-positive vessels was increased in stroma surrounding VEGF-C-positive prostatic carcinoma cells. These results suggest that the expression of VEGF-C in prostatic carcinoma cells is implicated in the lymph node metastasis. © 1999 Cancer Research Campaign


*British Journal of Cancer* (1999) **80**(1/2), 309–313
© 1999 Cancer Research Campaign
Article no. bjoc.1998.0356

### Vascular endothelial growth factor-C expression in human prostatic carcinoma and its relationship to lymph node metastasis

**T Tsurusaki[1,2], S Kanda[1], H Sakai[1], H Kanetake[1], Y Saito[1], K Alitalo[3] and T Koji[2]**

Departments of [1]Urology and [2]Histology and Cell Biology, Nagasaki University School of Medicine, 1-7-1 Sakamoto, Nagasaki 852-8102, Japan; [3]Molecular/Cancer Biology Laboratory, Haartman Institute, University of Helsinki, PL21, 00014 Helsinki, Finland

**Summary** Lymph node dissemination is a major prognostic factor in human cancer. However, the molecular mechanisms underlying lymph node metastasis are poorly understood. Recently, vascular endothelial growth factor-C (VEGF-C) was identified as a ligand for VEGF receptor-3 (VEGFR-3/Flt-4) and the expression of VEGFR-3 was found to be highly restricted to the lymphatic endothelial cells. In this report, we investigated the expression of VEGF-C and VEGFR-3 in human prostatic carcinoma tissue by using in situ hybridization and immunohistochemical staining respectively. Expression of VEGF-C mRNA in prostatic carcinoma was significantly higher in lymph node-positive group than in lymph node-negative group. In addition, the number of VEGFR-3-positive vessels was increased in stroma surrounding VEGF-C-positive prostatic carcinoma cells. These results suggest that the expression of VEGF-C in prostatic carcinoma cells is implicated in the lymph node metastasis.

**Keywords:** prostatic carcinoma; vascular endothelial growth factor-C; VEGF receptor-3; lymph node metastasis

Prostatic carcinoma is one of the major cancers in men. It is known that the progression of the disease is highly associated with metastasis to the bone and the lymph nodes (Slack et al, 1986). The major therapy for extensive disease is androgen deprivation therapy, whereas there are several ways to treat localized (non-metastatic) prostatic cancer, including anti-androgen therapy, radical prostatectomy, radiotherapy and cryotherapy. The prognosis is dependent on the presence of lymph node metastasis (Epstein et al, 1996). However, the molecular mechanisms underlying lymph node metastasis are poorly examined.

Vascular endothelial growth factor (VEGF)/vascular permeability factor (VPF) belongs to the platelet-derived growth factor (PDGF)/VEGF family, which is known as a potent inducer of angiogenesis (Dvorak et al, 1995; Ferrara and Davis-Smyth, 1997). VEGF receptor-1 (VEGFR-1; Flt-1) and VEGFR-2 (Flk-1/KDR) are receptor tyrosine kinases for VEGF/VPF (Ferrara and Davis-Smyth, 1997). These receptors are found to be largely expressed on the vascular endothelial cells. VEGFR-2 has an ability to transduce signals for biological responses leading to angiogenesis, including proliferation and migration of endothelial cells in vitro, whereas the significance of Flt-1 was unclear (Waltenberger et al, 1994). Recently, Flt-1 was reported to transduce signals for migration and activation of monocytes (Barleon et al, 1996; Clauss et al, 1996). Gene targeting of VEGFR-1 and VEGFR-2 results in the fetal death associated with the lack of normal vasculature in utero (Fong et al, 1995; Shalabi et al, 1995), indicating that VEGF receptors are important for vascular development. Recently, new members of the VEGF/VPF family, denoted VEGF-C and -D, were cloned (Joukov et al, 1996; Achen et al, 1998). Receptors for VEGF-C and -D are VEGFR-3 (Flt-4) (Pajusola et al, 1992) and VEGFR-2. VEGFR-2 has an affinity for VEGF/VPF, whereas VEGFR-3 can bind specifically to VEGF-C and -D. The distribution of VEGFR-3 is highly restricted to the lymphatic endothelial cells (Kaipainen et al, 1995), suggesting that VEGFR-3 is one of the specific markers for lymphatic endothelial cells. VEGF-C-transgenic mice revealed that an increase in the diameter of lymphatic vessels was the major effect of VEGF-C overexpression instead of an increase in number of vessels (Jeltsch et al, 1997). In contrast, VEGF-C-coated thermanox disks markedly induced lymphangiogenesis (increase in number of lymphatic vessels) in the differentiated avian chorioallantoic membrane (Oh et al, 1997). These results suggest differences in the effects of VEGF-C on embryonic and mature lymphatic vessels.

In this study, we examined the expression of VEGF-C in human prostatic carcinoma tissue and found that there was a significant correlation between the expression of VEGF-C and lymph node dissemination. Additionally, the number of VEGFR-3-positive vessels was increased in the surrounding stromal tissue of VEGF-C positive prostatic carcinoma cells. These results indicate that the determination of VEGF-C expression in prostatic carcinoma tissue would be useful to predict lymph node metastasis and suggest a role for VEGF-C in lymphatic metastasis.

*Received 8 June 1998*
*Revised 28 September 1998*
*Accepted 21 October 1998*

*Correspondence to:* S Kanda

### MATERIALS AND METHODS

### Tissue collection and preparation, and patients' characteristics

Prostate cancer tissues from 26 patients were used in this study. All tissue specimens were obtained by 16- or 18-gauge needle transperineal biopsies under informed consent prior to treatment.

Tissue specimens for in situ hybridization (ISH) were fixed in 10% neutral buffered formalin and embedded in paraffin, and some specimens for immunohistochemistry (IHC) were kept as fresh frozen sections. Histological grade was determined in each sample by using the Gleason grading system (Gleason et al, 1977) and the tissue area containing the primary grade site (predominant) was selected for further investigation. Serum prostatic specific antigen (PSA) was measured prior to the biopsy by using Tandem R assay. Patients were examined for detection of metastasis with chest X-ray, abdominal ultrasonography, computerized tomography scanning, magnetic resonance imaging and bone scintigraphy, and clinical stage was assessed by the 1992 TNM system (Hermanek and Sobin, 1992). Patients were divided into three groups: group 1 consisted of seven cases with clinically localized, well-differentiated (Gleason score 2–4) prostatic adenocarcinoma; group 2 consisted of ten cases with bone metastases, which are thought to be the metastatic sites via blood vessels; and group 3 consisted of nine cases with both bone and lymph node metastases. The mean ages of patients in each group were 74.0, 75.0 and 69.6 years old, respectively, and the differences were not statistically significant. There was no statistical difference in Gleason score and T stage between group 2 and group 3.

### Preparation of oligo-DNA probes

A 40-base sequence complementary to VEGF-C mRNA was selected. The antisense and sense sequences were synthesized together with two and three TTA repeats at the 5′- and 3′-ends of sequences for the T-T dimer formation. The sequences of antisense and sense probes used in this study were 5′-TTATTATGAGGTAGCTCGTGCTGGTGTTCATGCACT-GCAGCCCCTCACTATATTATTATT-3′ and 5′-TTATTAATAGT-GAGGGGCTGCAGTGCATGAACACCAGCACGAGCTACCT CAATTATTATT-3′, respectively. A computer-assisted search (GenBank Rel. 95.0, 1996) of the above antisense sequence, as well as the sense sequence, showed no significant homology with any known sequences. The T-T dimer, which is used to provide a hapten for antibody recognition of the ISH probes (Koji et al, 1996), was introduced into oligo-DNAs by UV irradiation (254 nm) with a dose of 12 000 J $m^{-2}$. The generation of T-T dimer was verified immunochemically by using mouse monoclonal anti-T-T IgG (Kyowa Medex, Tokyo, Japan).

### Dot-blot hybridization

Various amounts of the sense oligo-DNA between 1 pg and 10 ng were spotted onto nitrocellulose membranes that had been soaked in 20 × suline–sodium citrate (SSC) [1 × SSC; 0.015 M sodium citrate, pH 7.0 supplemented with 0.15 M sodium chloride (NaCl)]. After air-drying, the filters were baked at 80°C for 2 h, followed by the incubation with prehybridization medium containing 10 mM Tris-HCl, pH 7.4, 0.6 M NaCl, 1 mM EDTA, 1 × Denhardt's solution, 500 µg $ml^{-1}$ yeast tRNA, 250 µg $ml^{-1}$ salmon testicular DNA (Sigma, St Louis, MO, USA) and 40% (v/v) deionized formamide at 37°C for 2 h. The membranes were then incubated with 1 µg $ml^{-1}$ T-T dimerized oligo-DNA probe in hybridization medium containing 10 mM Tris-HCl (pH 7.4), 0.6 M NaCl, 1 mM EDTA, 1 × Denhardt's solution, 250 µg $ml^{-1}$ yeast tRNA, 125 µg $ml^{-1}$ salmon testicular DNA, 10% dextran sulphate and 40% deionized formamide at 37°C overnight. After washing with 2 × SSC, the membranes were stained immunochemically with anti-T-T IgG as described previously (Koji et al, 1996) and the signals were visualized by the incubation with 0.1 M phosphate buffer, pH 7.5, containing 0.15 mg $ml^{-1}$ 3,3′-diaminobenzidine-4 hydrochloric acid (HCl) (DAB), 0.01% hydrogen peroxide ($H_2O_2$), 0.025% $CoCl_2$ and 0.02% $NiSO_4(NH_4)_2SO_4$ (Adams, 1981).

### ISH for cultured cells

Human prostatic adenocarcinoma cell lines, PC-3, DU145 and LNCap cells were grown in Ham's F-12 medium supplemented with 10% heated-inactivated fetal bovine serum (Life Tech. Oriental, Tokyo, Japan). For ISH, cells were seeded into wells of 8-well Lab-Tek chamber glass slides (Nalge Nunc International, Naperville, IL, USA) and cultured overnight. ISH of these cells was performed according to the method described previously (Koji et al, 1996; Tsurusaki et al, 1998). Briefly, cells grown on glass slides were sequentially incubated with 0.2 N HCl for 20 min, 0.2% Triton X-100 in phosphate-buffered saline (PBS) for 10 min, and 2 µg $ml^{-1}$ proteinase K at 37°C for 20 min. After fixation with 4% paraformaldehyde in PBS for 5 min, the cells were immersed in PBS containing 2 mg $ml^{-1}$ glycine for 30 min and kept in 4 × SSC containing 40% deionized formamide until use for hybridization. Hybridization was carried out at 37°C overnight with 2 µg $ml^{-1}$ T-T dimerized antisense oligo-DNA for VEGF-C dissolved in hybridization medium. The cells were washed with 50% formamide in 2 × SSC for 5 h followed by washing with PBS, and were then incubated with anti-T-T dimer monoclonal antibody followed by the incubation with peroxidase-conjugated anti-mouse IgG. Peroxidase bound to tissue sections was visualized as described previously (Koji et al, 1996; Tsurusaki et al, 1998). Hybridization signals were considered positive when accumulated black deposits were seen in individual cells. When the signal was similar to that of the negative control, the sample was regarded as 'negative'. 'Weak positive' staining was assigned when the signals were present, but only localized to the perinuclear area. 'Strong positive' staining represented dense and clear signals throughout the cytoplasm.

### ISH for prostatic carcinoma specimens

Deparaffinized and rehydrated biopsy specimens were sequentially treated with 0.3% $H_2O_2$ in methanol for 15 min, 0.2 N HCl for 20 min and 100 µg $ml^{-1}$ proteinase K at 37°C for 15 min. ISH for biopsy specimens was performed according to the method discribed in ISH for cultured cells.

### Control experiments for ISH

To evaluate the specificity of VEGF-C mRNA signals, a panel of control experiments were conducted on sections adjacent to the malignant area (Koji et al, 1996; Tsurusaki et al, 1998). A number of consecutive tissue sections were hybridized with T-T dimerized oligo-DNA complementary to 28S rRNA as a positive control (Yoshii et al, 1995) and also T-T dimerized VEGF-C sense oligo-DNA as a negative control in every run. Another group of tissue sections was hybridized with the VEGF-C antisense probe in the presence of an excess amount (50-fold) of either homologous or non-homologous unlabelled oligo-DNA probe, to confirm the sequence specificity of the signals. To eliminate possible involvement of

 *© Cancer Research Campaign 1999*

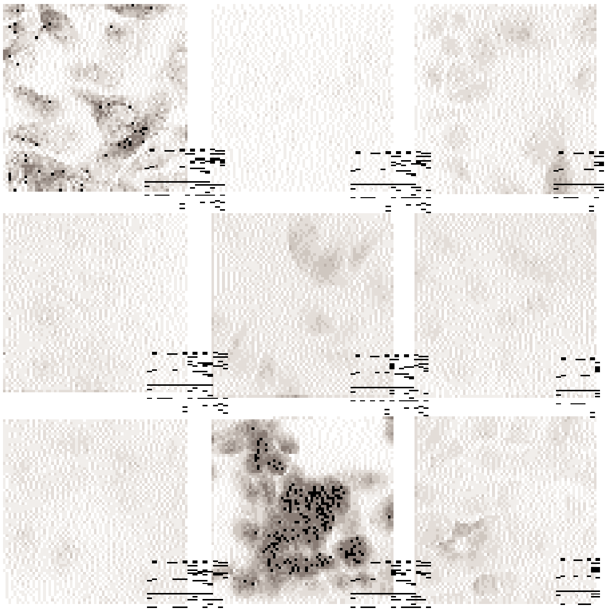

**Figure 1** VEGF-C mRNA expression in human prostatic carcinoma cell lines, PC-3 (**A**–**G**), LNCaP (**H**) and DU-145 (**I**) by using the non-radioactive in situ hybridization method. (**A**) 28S rRNA antisense probe; (**B**) VEGF-C sense probe; (**C, H, I**) VEGF-C antisense probe; (**D**) competition with excess amount (50-fold) of unlabelled probe; (**E**) competition with excess amount (50-fold) of non-homologous unlabelled oligo-DNA; (**F**) hybridization with antisense probe followed by washing at excessively high stringency; (**G**) treatment with RNase-A before hybridization with VEGF-C antisense probe. Magnification × 400

proteins and DNA in signal formation, some sections were digested with 100 μg ml$^{-1}$ of ribonuclease-A (RNase-A) at 37°C for 60 min before the post-fixation step. In some sections, excessively stringent washing conditions (50% formamide in 2 × SSC at 37°C for 5 h and 50% formamide in 0.1 × SSC at 45°C for 30 min) were applied after hybridization with the VEGF-C antisense probes.

### IHC for VEGFR-3

The monoclonal antibody raised against the extracellular domain of human VEGFR-3 (mouse $IgG_1$) (Jussila et al, 1998) was used. The antibody could recognize VEGFR-3 expressed in tissues prepared as frozen sections. Fresh frozen prostatic tissue sections from six patients (two sections each from VEGF-C-negative, VEGF-C weakly positive and VEGF-C strongly positive cancer tissues) were fixed with acetone at 4°C and treated with 5% normal goat serum in 1% bovine serum albumin in PBS. Then the sections were incubated with 2 μg ml$^{-1}$ monoclonal antibody at room temperature overnight and then with horseradish peroxidase (HRP) conjugated goat anti-mouse IgG for 60 min. Peroxidase was visualized with DAB and $H_2O_2$, and the sections were counterstained with methyl green and mounted. The consecutive sections were incubated with 2 μg ml$^{-1}$ normal mouse IgG in place of the specific monoclonal antibody and used as a negative control.

### Statistical analysis

The $\chi^2$ test was used to analyse the relationship of VEGF-C mRNA expression between groups 1 + 2 and 3.

**Table 1** Summary of VEGF-C mRNA expression in primary prostatic carcinoma specimens determined by in situ hybridization

| | Group 1 | Group 2 | Group 3 |
|---|---|---|---|
| Gleason sum | 4 ± 0 | 8.2 ± 1.1 | 8.9 ± 0.9 |
| PSA (ng ml$^{-1}$) | 18.3 ± 10.9 | 213.2 ± 393.9 | 622.3 ± 799.4 |
| T stage | 2.3 ± 0.5 | 3.5 ± 0.5 | 3.0 ± 0.5 |
| Metastatic sites | None | Bone | Bone with lymph nodes |
| VEGF-C mRNA expression | | | |
| Negative | 4 | 6 | 0 |
| Weakly positive | 1 | 3 | 3 |
| Strongly positive | 2 | 1 | 6 |

Formalin-fixed, paraffin-embedded tissues were hybridized with T-T dimerized antisense probes for VEGF-C and hybridized probes were visualized by using anti-T-T antibody followed by peroxidase reaction. No staining of tumour cells is indicated as 'negative'. 'Weakly positive' indicates a positive staining restricted to the perinuclear area, and 'strongly positive' indicates diffuse cytoplasmic staining in the tumour cells. Group 3 (with lymph node metastasis) demonstrated significantly stronger VEGF-C mRNA expression than other groups (without lymph node metastasis; $P = 0.0083$, by the $\chi^2$ test).

### RESULTS

### Dot-blot hybridization with T-T dimerized synthetic oligo-DNA

To examine the sensitivity of synthesized antisense oligo-DNA, VEGF-C sense oligo-DNA on a nitrocellulose membrane was hybridized with T-T dimerized VEGF-C antisense oligo-DNA or T-T dimerized VEGF-C sense oligo-DNA. One picogram of the sense DNA was detected with the antisense probe, while the sense oligo-DNA detected no signal (data not shown). These results indicate that the antisense probe was specific and had adequate sensitivity to be used in ISH studies.

### VEGF-C mRNA expression in cultured human prostatic carcinoma cells assessed by ISH

To certify the quality of T-T dimerized VEGF-C antisense oligo-DNA in ISH study, expression of mRNA for VEGF-C in PC-3, LNCaP and DU145 cells was examined. VEGF-C mRNA was expressed in the cytoplasmic area of more than 90% of these cells. In LNCaP cells, high expression of VEGF-C was observed, and DU145 cells were found to express low amount of VEGF-C mRNA. To confirm the sequence specificity of the VEGF-C mRNA signal in these cell lines, we conducted various control experiments. A representative set of control experiments on PC-3 was shown in Figure 1A, B and D–G. When cells were hybridized with the T-T dimerized oligo-DNA complementary to 28S rRNA, signals were detected in the cytoplasmic area as well as in nucleoli (Figure 1A), whereas no staining was found with T-T dimerized VEGF-C sense probe (Figure 1B). When cells were hybridized with VEGF-C antisense probe in the presence of excess amount of the homologous unlabelled oligo-DNA, cytoplasmic signal was much weaker (Figure 1D). On the other hand, the VEGF-C mRNA staining was not altered by the presence of excess amount of non-homologous unlabelled oligo-DNA (Figure 1E). When cells were washed at excessively high stringency or were digested with RNase-A, a remarkable decrease in the staining was observed (Figure 1F, G).

© Cancer Research Campaign 1999

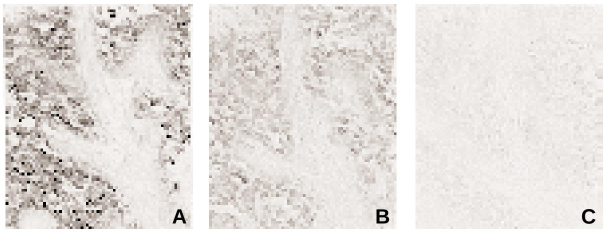

**Figure 2** VEGF-C mRNA expression of a grade 5 prostatic carcinoma tissue visualized by using the non-radioactive in situ hybridization method. (**A**) 28S rRNA antisense probe used as a positive control; (**B**) VEGF-C antisense probe; (**C**) VEGF-C sense probe used as a negative control. Magnification x 200

### VEGF-C mRNA expression in human prostatic carcinoma tissues

Twenty-six human prostatic carcinoma samples were examined for the expression of VEGF-C mRNA by ISH. Sixteen specimens out of 26 (61.5%) were estimated as positive, in which the cancer epithelial cells were positive for VEGF-C mRNA, while other types of cells, such as basal cells, stromal cells, endothelial cells and vascular smooth muscle cells, were negative for VEGF-C expression. The staining for VEGF-C mRNA was localized to the cytoplasmic area of epithelial cells. A typical staining is shown in Figure 2. In group 1, three specimens out of seven (42.9%) were estimated as positive (two strongly positive and one weakly positive). In group 2, four specimens out of ten (40%) were positive for VEGF-C mRNA, and in group 3, all nine specimens (100%) were positive (Table 1). Group 3 (with lymph node metastases) demonstrated significantly higher VEGF-C mRNA expression than other groups (without lymph node metastases) ($P = 0.0083$) and no significant difference was observed between localized disease and advanced disease (group 1 vs group 2). These data demonstrate that the expression of VEGF-C mRNA in prostatic carcinoma cells is closely associated with the presence of lymph node metastasis.

### VEGFR-3 protein expression assessed by IHC

Receptors for VEGF-C were identified as VEGFR-2 and -3 (Joukov et al, 1996). Since the expression of VEGFR-3 is highly restricted to lymphatic endothelial cells (Kaipainen et al, 1995), it has been suggested that VEGF-C is involved in lymphangiogenesis (increase in the number of lymphatic vessels) in differentiated tissues. To examine the role of VEGF-C expression in prostatic carcinoma, VEGFR-3 expression was studied in two fresh frozen sections from each of VEGF-C-negative, VEGF-C weakly positive and VEGF-C strongly positive groups by IHC. Two specimens in each group showed representative results and the typical tumour areas from each group are shown in Figure 3. In the VEGF-C mRNA-negative specimens, VEGFR-3-positive vessels were hardly observed, as shown in Figure 3A. However, in the VEGF-C mRNA-positive specimens, the number of VEGFR-3-positive vessels was clearly increased in the stromal tissues surrounding tumour nests, as shown in Figure 3B and 3C. The VEGFR-3-positive vessels were hardly observed in the tumour nests and none of the VEGFR-3-positive channels contained red blood cells.

### DISCUSSION

In this study, we demonstrated that the expression of VEGF-C in human prostatic carcinoma cells was significantly correlated with the presence of lymph node metastasis. In addition, VEGFR-3-positive vessels (possibly lymphatic vessels), which were observed only in tumour stromal tissue, were increased in density when VEGF-C was expressed in the prostatic carcinoma cells, indicating a paracrine relationship between VEGF-C expressed in prostatic cancer cells and its receptor, VEGFR-3, expressed in adjacent stromal tissue. This is the first demonstration of the association of VEGF-C expression and lymphatic dissemination of human carcinoma.

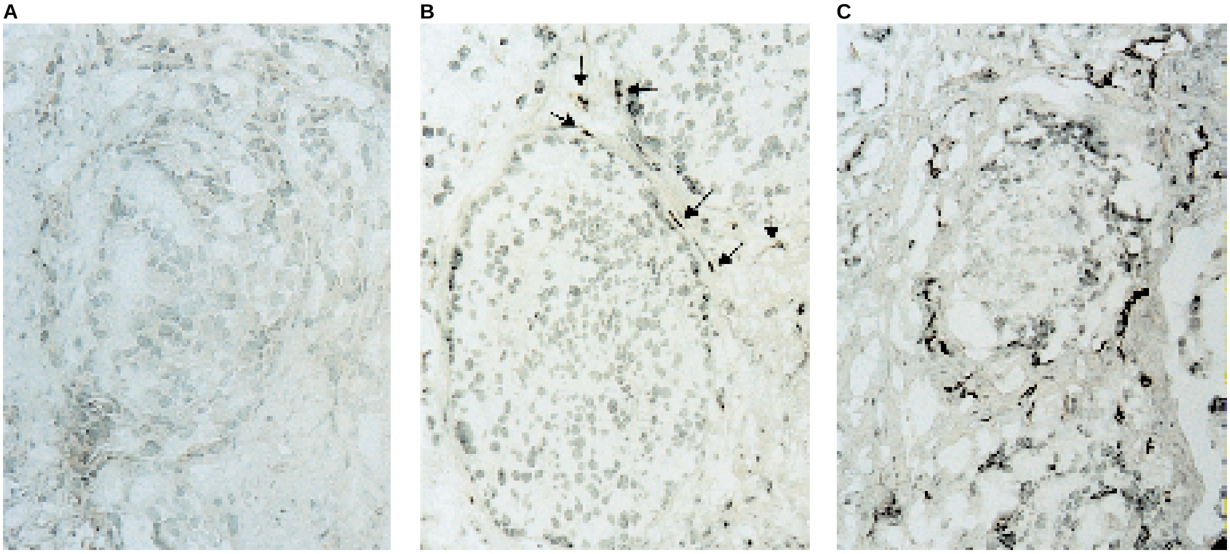

**Figure 3** VEGFR-3 expression in fresh frozen sections of prostate carcinoma tissue examined by immunohistochemistry. (**A**) VEGF-C mRNA-negative specimen; (**B**) in VEGF-C mRNA weakly positive specimen; (**C**) VEGF-C mRNA strongly positive specimen. Arrows in (**B**) show the positive staining of VEGFR-3. Magnification × 400

 *© Cancer Research Campaign 1999*

There is no definitive marker for the identification of lymphatic endothelial cells yet. According to recent studies (Jussila et al, 1998), the combination of anti-PAL-E and anti-CD31 immunostaining was useful to recognize lymphatic endothelial cells (PAL-E-negative/CD31-positive) and VEGFR-3 expression was mostly observed in lymphatic endothelial cells. Our results indicate that VEGFR-3 expression could be one of the good markers of lymphatic endothelial cells. In this study, VEGFR-3-positive cells were found only in connective tissue stroma surrounding tumour nests and none of the VEGFR-3-positive channels contained red blood cells. These findings suggest that VEGFR-3-positive cells are possibly lymphatic endothelial cells.

VEGF-C is a ligand for VEGFR-2 and -3. VEGFR-2 is expressed in both blood vessel and lymphatic endothelial cells. In the present study, we could not observe a remarkable difference of the blood vessel density between VEGF-C-positive and -negative prostatic carcinoma specimens (data not shown). Angiogenesis is stimulated by a variety of growth factors, including fibroblast growth factors, VEGF and transforming growth factor-β (Folkman and Shing, 1992). In prostatic carcinoma, many of these growth factors are known to be expressed (Steiner, 1993) and angiogenesis in prostatic carcinoma may be regulated by these growth factors rather than VEGF-C. In contrast, the expression of VEGFR-3 is highly restricted to lymphatic endothelial cells. The role of the signal transduction pathway via VEGFR-3 in the biological responses of lymphatic endothelial cells has not been clarified yet. However, the present data is compatible with the view that VEGF-C stimulates lymphangiogenesis in vivo. In transgenic mice, overexpression of VEGF-C resulted in the increase in diameter of lymphatic vessels, whereas an increase in the number of vessels was not observed (Jeltsch et al, 1997). In the differentiated chorioallantoic membrane, VEGF-C was stimulatory also for in vivo lymphangiogenesis (Oh et al, 1997). The difference of VEGF-C functions between developing and mature tissues may be due to the presence of specific signal transduction pathways or an unidentified specific receptor for VEGF-C, which could be expressed only in mature lymphatic endothelial cells and could be necessary for the increase in lymphatic vessels.

In the present study, some clinically lymph node-negative prostatic carcinoma specimens also showed VEGF-C expression. It is possible that VEGF-C-positive prostatic carcinoma patients without clinical lymph node metastasis in the present study might have lymph node metastasis at microscopic level. Nevertheless, more importantly, the expression of VEGF-C was significantly higher in lymph node-positive patients than in node-negative patients, suggesting that the examination of VEGF-C expression in prostatic carcinoma tissue may be an indicator of the presence of lymph node metastasis.

### ACKNOWLEDGEMENTS

We are grateful to Dr Shin-ichi Kiyokawa for the preparation of cryostat sections. We also thank Takumi Shimogama and Miki Yoshimoto for their outstanding help. This work was supported by a grant-in-aid for scientific research (A) (No. 06404059) from the Ministry of Education, Science, Sports and Culture, Japan.

### REFERENCES

Achen MG, Jeltsch M, Kukk E, Makinen T, Vitali A, Wilks AF, Alitalo K and Stacker SA (1998) Vascular endothelial growth factor D (VEGF-D) is a ligand for the tyrosine kinases VEGF receptor-2 (Flk1) and VEGF receptor-3 (Flt-4). *Proc Natl Acad Sci USA* **95**: 548–553

Adams JC (1981) Heavy metal intensification of DAB-based HRP reaction product. *J Histochem Cytochem* **29**: 775

Barleon B, Sozzani S, Zhou D, Weich H, Mantovani A and Marmé D (1996) Migration of human monocytes in response to vascular endothelial growth factor (VEGF) is mediated via the VEGF receptor flt-1. *Blood* **87**: 3336–3343

Clauss M, Weich H, Breier G, Knies U, Rock W, Waltenberger J and Risau W (1996) The vascular endothelial growth factor receptor Flt-1 mediates biological activities. Implications for a functional role of placenta growth factor in monocyte activation and chemotaxis. *J Biol Chem* **271**: 17629–17634

Dvorak HF, Brown LF, Detmar M and Dvorak AM (1995) Vascular permeability factor/vascular endothelial growth factor, microvascular permeability, and angiogenesis. *Am J Pathol* **146**: 1029–1039

Epstein JI, Partin AW, Sauvageot J and Walsh PC (1996) Prediction of progression following radical prostatectomy: a multivariate analysis of 721 men with long-term follow-up. *Am J Surg Pathol* **20**: 286–292

Ferrara N and Davis-Smyth T (1997) The biology of vascular endothelial growth factor. *Endocrine Rev* **18**: 4–25

Folkman J and Shing Y (1992) Angiogenesis. *J Biol Chem* **267**: 10931–10934

Fong G-H, Rossant J, Gertenstein M and Breitman M (1995) Role of Flt-1 receptor tyrosine kinase in regulation of assembly of vascular endothelium. *Nature* **376**: 66–67

Gleason DF (1977) Histologic grading and clinical staging of prostatic carcinoma. In *Urologic Pathology: Prostate*, Tannenbaum M (ed), pp. 171–198. Lea & Febiger, Philadelphia

Jeltsch M, Kaipainen A, Joukov V, Meng X, Lakso M, Rauvala H, Swartz M, Fukumura D, Jain RK and Alitaro K (1997) Hyperplasia of lymphatic vessels in VEGF-C transgenic mice. *Science* **276**: 1423–1425

Joukov V, Pajusola K, Kaipainen A, Chilov D, Lahtinen I, Kukk E, Saksela O, Kalkkinen N and Alitalo K (1996) A novel vascular endothelial growth factor, VEGF-C, is a ligand for the Flt-4 (VEGFR-3) and KDR (VEGFR-2) receptor tyrosine kinases. *EMBO J* **15**: 290–298

Jussila L, Valtola R, Partanen TA, Salven P, Heikkilä P, Matikainen M-T, Renkonen R, Kaipainen A, Detmar M, Tschachler E, Alitalo R and Alitalo K (1998) Lymphatic endothelium and Kaposi's sarcoma spindle cells detected by antibodies against the vascular endothelial growth factor receptor-3. *Cancer Res* **58**: 1599–1604

Kaipainen A, Korhonen J, Mustonen T, van Hinsbergh VWM, Fang G-H, Dumont D, Beitman M and Alitalo K (1995) Expression of the fms-like tyrosine kinase 4 gene becomes restricted to lymphatic endothelium during development. *Proc Natl Acad Sci USA* **92**: 3566–3570

Koji T and Nakane PK (1996) Recent advances in molecular histochemical techniques: in situ hybridization and southwestern histochemistry. *J Electron Microsc* **45**: 119–127

Oh S-J, Jeltsch M, Birkenhäger R, McCarthy JEG, Weich HA, Christ B, Alitaro K and Wilting J (1997) VEGF and VEGF-C: Specific induction of angiogenesis and lymphangiogenesis in the differentiated avian chorioallantoic membrane. *Dev Biol* **188**: 96–109

Pajusola K, Aprelikova O, Korhonen J, Kaipainen A, Pertovaara L, Alitalo R and Alitalo K (1992) FLT4 receptor tyrosine kinase contains seven immunoglobulin-like loops and is expressed in multiple human tissues and cell lines. *Cancer Res* **52**: 5738–5743

Shalabi F, Rossant J, Yamaguchi TP, Gertenstein M, Wu X-F, Breitman ML and Schuh AC (1995) Failure of blood island formation and vasculogenesis in Flk-1 dificient mice. *Nature* **376**: 62–66

Slack NH, Lane WW, Priore RL and Murphy GP (1986) Prostatic cancer treated at a categorical center, 1980–1983. *Urology* **27**: 205–213

Steiner MS (1993) Role of peptide growth factors in the prostate: a review. *Urology* **42**: 99–110

Tsurusaki T, Koji T, Sakai H, Kanetake H, Nakane PK and Saito Y (1998) Cellular expression of beta-microseminoprotein (β-MSP) mRNA and its protein in untreated prostate cancer. *Prostate* **35**: 109–116

Waltenberger J, Claesson-Welsh L, Siegbahn A, Shibuya M and Heldin C-H (1994) Different signal transduction properties of KDR and Flt-1, two receptors for vascular endothelial growth factor. *J Biol Chem* **269**: 26988–26995

Yoshii A, Koji T, Ohsawa N and Nakane PK (1995) In situ localization of ribosomal RNAs in a reliable reference for hybridizable RNA in tissue sections. *J Histochem Cytochem* **43**: 321–327

© Cancer Research Campaign 1999
